# Dispersal of the Invasive Pasture Pest *Heteronychus arator* into Areas of Low Population Density: Effects of Sex and Season, and Implications for Pest Management

**DOI:** 10.3389/fpls.2016.01278

**Published:** 2016-08-26

**Authors:** Sarah Mansfield, Philippa J. Gerard, Mark R. H. Hurst, Richard J. Townsend, Derrick J. Wilson, Chikako van Koten

**Affiliations:** ^1^AgResearch LincolnChristchurch, New Zealand; ^2^Bio-Protection Research Centre, Lincoln UniversityLincoln, New Zealand; ^3^AgResearch RuakuraHamilton, New Zealand

**Keywords:** black beetle, insect dispersal, mark–release–recapture, pitfall traps, ryegrass

## Abstract

African black beetle, *Heteronychus arator* (Scarabaeidae), is an exotic pest of pastures in northern New Zealand. Both adults and larvae feed on pasture grasses. Adults disperse by walking (short range) or flying (long range). Dispersal flights are triggered by warm night temperatures in spring and autumn. Short range adult dispersal in search of mates, food or oviposition sites is poorly understood. This study investigated walking activity of *H. arator* adults over three seasons in New Zealand pastures. Adult walking activity was monitored using pitfall traps along fence lines and in pasture plots on a dairy farm in Waikato, New Zealand, in spring 2013, spring 2014, and autumn 2015. Beetle populations were reduced by application of a biopesticide bait to compare walking activity between treated and control plots for up to 26 days post-treatment. Marked beetles were released into the pasture plots to measure the distance traveled by recaptured individuals. Trap catches along the fence lines were correlated with air temperatures in 2013. Trap catches were male biased in spring 2014 compared with autumn 2015. Trap numbers in the control plots were nearly double that of treated plots in both seasons. More beetles were caught in the pitfall traps at the edges of the treated plots than in the center. Trap catches were consistent throughout the control plot in spring 2014, but in autumn 2015 more beetles were caught in the center of the control plot than at the edges. Few marked beetles were recaptured with dispersal rates estimated as <0.5 m per day. Warmer temperatures encouraged short range dispersal in *H. arator*. Males were more active than females during the spring mating season. Edge effects were strong and should be considered in the design of field experiments.

## Introduction

The African black beetle, *Heteronychus arator* (Scarabaeidae), was first discovered in New Zealand in 1937 (Chapman, 1984). The distribution of this subtropical species in New Zealand is limited by climate (Watson, 1979) to Northland, Waikato, Bay of Plenty and coastal areas of the northern North Island (Bell et al., 2011). It has become a major pest of pastures and maize crops in northern New Zealand and is also a pasture pest in parts of Australia (Bulinski et al., 2006). In both countries, *H. arator* overwinters as an adult with mating and oviposition taking place in spring. Larvae are present during summer followed by pupation and adult emergence in autumn (Chapman, 1984; Matthiessen and Ridsdill-Smith, 1991). Adults feed on plant stems and roots just below the soil surface, while larvae feed below ground on detritus and plant roots, with third instar larvae the most damaging life stage (Ball et al., 1997). *H. arator* also damages horticultural crops such as potato (Matthiessen and Learmonth, 1995) and even eucalypt seedlings (Bulinski et al., 2006).

The primary strategy for controlling *H. arator* in New Zealand pastures is sowing ryegrass varieties with associated endophytes that confer resistance to herbivory (Thom et al., 2014). These varieties limit *H. arator* populations, but outbreaks still occur, particularly during warmer La Niña years (Bell et al., 2011; Gerard et al., 2013). No insecticides are registered for use against *H. arator* in New Zealand pastures, and results of insecticide experiments in Australia and New Zealand have been mixed (Matthiessen and Learmonth, 1995; Bulinski and Matthiessen, 2002; Bulinski et al., 2006; Eden et al., 2011). A common outcome for experiments conducted in open field plots has been short-term *H. arator* mortality immediately after insecticide treatment, but no associated reduction in damage and/or no reduction in subsequent populations. Two possible explanations have been proposed: The first is short insecticide persistence and difficulty achieving contact between the insecticide and beetle adults or larvae. The second is *H. arator* re-colonization of treated plots from surrounding areas (Matthiessen and Learmonth, 1995; Bulinski and Matthiessen, 2002; Eden et al., 2011). This study focused on *H. arator* dispersal.

Adult *H. arator* disperse by flying and walking. Dispersal flights occur primarily in autumn when the beetles are reproductively immature (Watson, 1979; Matthiessen and Learmonth, 1998; Hardwick, 2004). Newly sown or renovated pastures are colonized by dispersal flights (Hardwick, 2004) and flights are probably most important for dispersal between paddocks and farms. Walking is, however, more relevant for field plot experiments, which are usually on a smaller spatial scale than whole paddocks. For example, adults walk to aggregate around patches of favored food plants within paddocks (King et al., 1981).

To understand short range dispersal of *H. arator* requires measurement of population density and adult activity. Population density can be measured by sampling either cores or spade squares of soil to count the number of individuals and converting them to numbers/m^2^ (Watson et al., 1980). When *H. arator* densities are high (>50 adults/m^2^), this method is sufficiently sensitive to measure population responses to treatments, but when populations are low (typically 0–25 adults/m^2^; Gerard et al., 2013), impractically high sample numbers are required to assess treatment effects within field plot experiments. Pitfall traps are commonly used to measure dispersal and spatial patterns in populations of ground dwelling beetles (Matthiessen and Learmonth, 1998; Noronha and Cloutier, 1999; Negro et al., 2008; Elek et al., 2014). Pitfall traps also estimate relative density, but should be interpreted cautiously because the number of traps and their positions will affect capture rates (Winder, 2004). Mark–release–recapture (MRR) is another technique used to study dispersal in ground dwelling beetles (Klingenberg et al., 2010; Elek et al., 2014) that has not been tried for *H. arator* in New Zealand. Here, we use pitfall traps to investigate (1) the relationship between adult *H. arator* walking activity and temperature, (2) movement of adults into field plots after treatment with a biopesticide, and (3) distances traveled by individuals using MRR. A greater understanding of dispersal behavior in *H. arator* will assist with design and interpretation of field plot experiments so that treatment effects can be detected reliably.

## Materials and Methods

### Study Site and Trap Design

All experiments were conducted near Gordonton, New Zealand (-37.58, 175.28) using nine paddocks at a dairy farm with permanent pastures and consistently high populations of *H. arator*. The paddocks comprised mixtures of perennial ryegrass (*Lolium perenne*) and white clover (*Trifolium repens*) and were generally similar in terrain except that a runway for small aircraft was located in one paddock. Pitfall traps for all experiments consisted of plastic pots (approximately 100 mm diameter and depth), dug into the soil so the top of the cup was flush with the surface. Drainage holes (5 mm diameter) were drilled in the bottom of each pot and a small quantity of soil was put in the base of each pot to provide a refuge for trapped beetles.

### Temperature and African Black Beetle Activity (Spring 2013)

On August 30, 2013, five fence lines were selected adjacent to eight paddocks known to be infested with *H. arator*, and 20 pitfall traps were placed along each of the fence lines at 3–5 m intervals (trap lines 1–5). Placement of traps adjacent to the fences minimized the risk of damage from grazing livestock. Beetles were removed and counted from traps twice each week until November 13, 2013 (total of 21 sampling occasions). The mean number of beetles caught per trap for each fence line was then calculated for each sampling interval. Trap catch was log_e_-transformed prior to analysis. Daily temperature data collected at the nearest weather station (Ruakura Research Centre, approximately 14 km away from the study site) was obtained from the National Climate Database via the NIWA CliFlo website (NIWA, 2015). Daily temperatures were averaged across each sampling interval before analysis. The relationship between trap catches and maximum (Tmax), minimum (Tmin), and mean (Tmean) air temperatures, and minimum grass temperature (Tgrass), was then investigated using separate non-linear regressions (Minitab v.16). This avoided problems with collinearity between the temperature variables.

### African Black Beetle Dispersal into Areas of Low Density (Spring 2014 and Autumn 2015)

The spring trial ran from October 24 to November 17, 2014 and the autumn trial from April 15 to May 11, 2015 and the paddocks were grazed just before treatment application. Different paddocks were selected in spring and autumn to ensure sufficiently high African black beetle densities for detection of differences between the control and treated plots. In spring both plots were distanced from the paddock edges on all sides so that each plot had a similar area of surrounding untreated paddock. In autumn the control plot was approximately 5 m away from the paddock boundary on two sides, and was 10 m away from the treated plot. This plot placement was to avoid a microlight aircraft runway through the center of the paddock.

**FIGURE 1:**
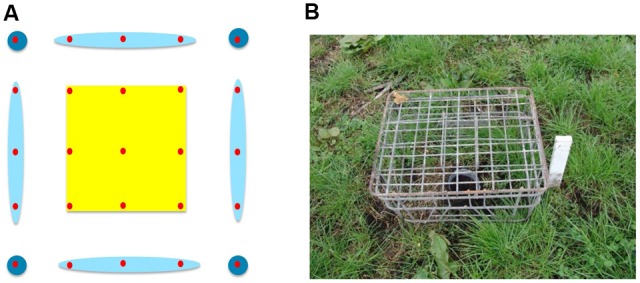
**(A)** Spatial grouping of the 25 pitfall traps (red circles) within each plot. The center (yellow) includes nine traps, the edges (light blue) had 12 traps, and there were four corner traps (dark blue); **(B)** A pitfall trap in the paddock protected from damage by birds with a wire crate.

In both trials, two 40 × 40 m open field plots were established side by side and 10 m apart. In each plot, a 10 × 10 m grid of pitfall traps was set up and traps in each grid were classified as corner, edge, or center (**Figure 1A**). Traps were protected by wire crates (45 × 36 × 23 cm) to stop birds raiding them (**Figure 1B**), and were emptied twice weekly for 24 days (spring) or 26 days (autumn) after treatment. One plot was the control and the other was treated with a prototype biopesticide bait to reduce beetle numbers (Hurst et al., 2011a,b; Hurst and Swaminathan, 2016) at a rate of 70 kg bait per ha, which equated to 11.2 kg bait per plot. Baits were distributed by hand evenly throughout the treated plot in each season.

All trapped beetles were taken back to the Ruakura Research Centre to be counted, sexed and checked for markers (see section 2.4). The sex ratio of the trapped beetles was compared using a χ^2^ test (Sigmaplot v.13). The number of beetles trapped was compared between treated and control plots, and between the three trap locations, using a generalised estimating equations (GEE) approach with a factor-specific negative binomial distribution and a log link function. The three factors were: treatment (treated or control), days after treatment (for seven sample dates) and trap location (corner, edge, or center). Two-way interactions between factors were included in the model: treatment^∗^day, treatment^∗^trap location, and day^∗^trap location. The GEE analysis used a first order autoregressive covariance structure to account for correlation among the seven sample dates (i.e., repeated measurements). This analysis was conducted using SAS v.9.3.

### Mark–Release–Recapture (Spring 2014 and Autumn 2015)

**FIGURE 2:**
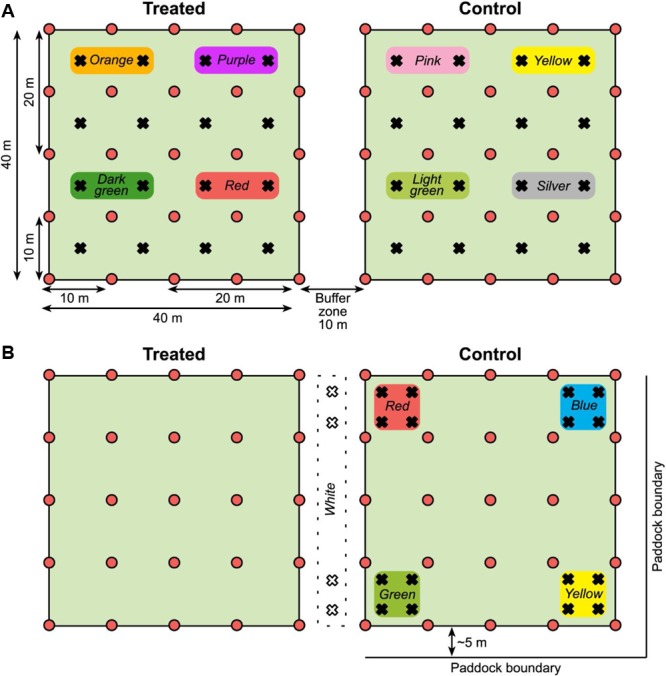
**(A)** Plot layout for the spring 2014 MRR experiment (not to scale). Pitfall traps shown as red circles and release points for marked beetles as black crosses. Color markers used on beetles released in each quadrant are shown. Both plots were in the middle of the paddock, distanced from the boundaries. **(B)** Plot layout for the autumn 2015 MRR experiment. Symbols and colors as stated for the spring; in autumn the control plot was approximately 5 m from the paddock boundary on two sides. Note that marked beetles were released only in the control plot and the buffer zone between plots in autumn.

In addition to the grid of pitfall traps, a MRR study was carried out. The intention was to measure the distances traveled by individual beetles to help interpret the general activity measured by trap captures across the plots. In spring live beetles (*n* = 768) were collected from pitfall traps placed along the fence lines. The day before the trial was set up, the beetles were divided into eight equal groups (*n* = 96); each group marked with a unique color (a patch of nail varnish on top of the prothorax). Each color group was assigned to one of four 20 × 20 m quadrants within each of the two 40 × 40 m plots (**Figure 2A**), and 24 beetles were released at each of four locations within each quadrant. Marked beetles were released by hand after the bait was applied to the treated plot. Beetles collected from the 10 × 10 m grid of traps were checked for color marks and their locations recorded so that the minimum distance traveled by individual beetles could be calculated.

The MRR study was repeated in autumn with some modifications, again using beetles collected from pitfall traps placed along paddock fence lines. We had observed deterioration of the nail varnish marks over time so there was concern that the lower than expected spring recapture rate [see Mark–Release–Recapture (Spring 2014 and Autumn 2015)] was due to mark loss as the beetles burrowed through soil. In autumn nail varnish was replaced with queen bee markers (small disks of colored plastic, Australian Entomological Supplies) that were glued to the beetles. Marked beetles were also released only in the control plot and in the buffer zone between plots, not in the treated plot (*n* = 96 beetles for each color group, **Figure 2B**) as there was limited availability of beetles.

The minimum distance traveled by each recaptured beetle in spring and autumn was calculated based on the distance between the trap where the marked beetle was captured and the nearest release point for beetles with that color mark. The sex ratio of marked beetles was 50:50 in both spring and autumn.

## Results

### Temperature and African Black Beetle Activity (Spring 2013)

**FIGURE 3:**
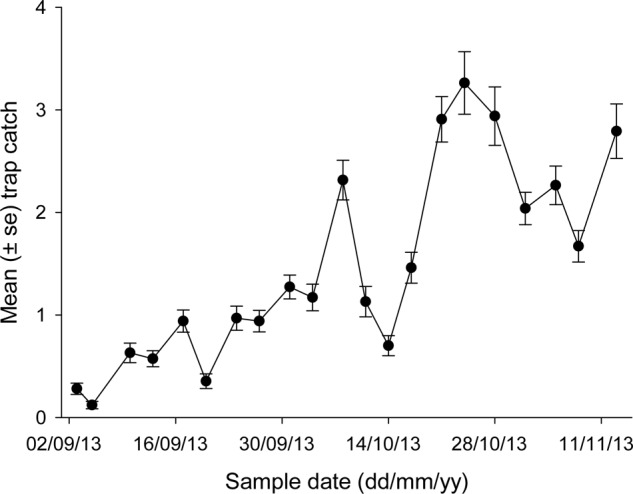
**Mean trap catch of African black beetles at each sample date for all five trap lines in spring 2013**.

**FIGURE 4:**
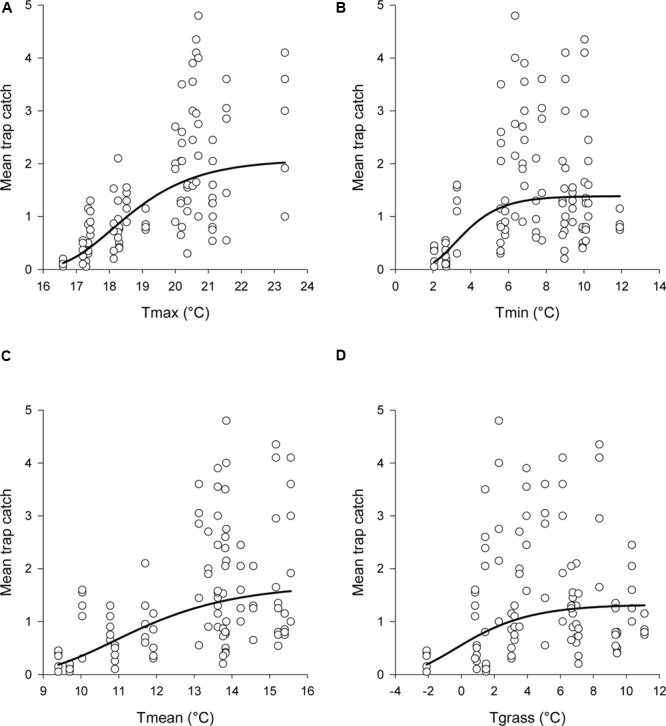
**Observed data and estimated relationships between mean trap catch and **(A)** Tmax, **(B)** Tmin, **(C)** Tmean, and **(D)** Tgrass**. Daily temperatures were averaged across each sampling interval. The non-linear regression model was: log_e_catch = a-exp(-b^∗^temperature + c). Estimated catches from the regressions were back transformed before graphing. Note that the *x*-axis scale differs between graphs.

Average trap catches varied noticeably during spring (**Figure 3**), ranging from <0.5 beetles per trap at the earliest sample dates to >3 beetles per trap on some dates in October. Peak catches were generally seen on October 24, although trap line 5 had been destroyed by calves on this date (the only mishap of note). The relationship between trap catch and temperature took the form:

$$\begin{array}{c} \log_{e}\mathrm{catch}\;=\;a\;-\;\exp(-\;b*\mathrm{temperature}+c) \end{array}$$

for each temperature variable examined (**Table 1**). This meant that trap catches increased toward an asymptote with increasing temperatures (**Figure 4**). Of the four temperature variables, the relationship with Tmax gave the closest fit to the data, although all four variables had a significant relationship with trap catch (**Table 1**).

**Table 1:** Non-linear regressions to estimate the relationships between mean trap catch and the four temperature variables.

Variable	Regression equation, *y* = log_e_catch, *x* = temperature	*P*	Variation (%) explained by model
Tmax	*y* = 0.7 - exp (- 0.7^∗^*x* + 12.7)	<0.001	60.1
Tmin	*y* = 0.3 - exp (- 0.7^∗^*x* + 2.4)	0.004	40.6
Tmean	*y* = 0.5 - exp (- 0.4^∗^*x* + 6.0)	0.01	40.2
Tgrass	*y* = 0.3 - exp (- 0.4^∗^*x* – 0.1)	0.01	18.4

*All relationships were statistically significant (*P* < 0.05).*

### African Black Beetle Dispersal into Areas of Low Density (Spring 2014 and Autumn 2015)

#### Spring 2014

**FIGURE 5:**
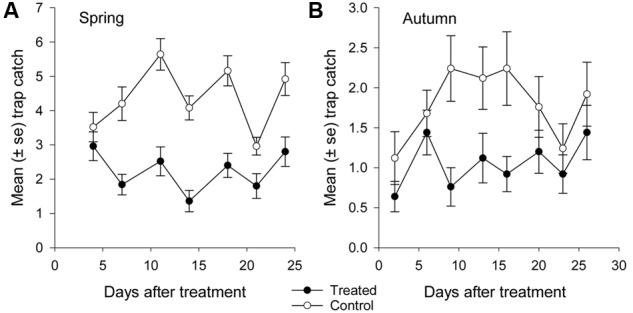
**Mean catch of African black beetles per pitfall trap in the treated and control plots in **(A)** spring 2014 (4–24 days after treatment) and **(B)** autumn 2015 (2–26 days after treatment)**.

**FIGURE 6:**
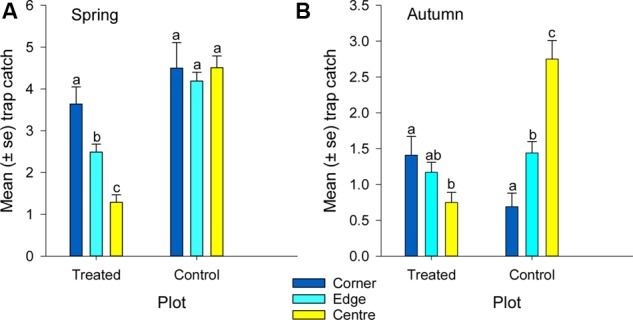
**Mean catch of African black beetles per pitfall trap from corner to edge to center of the treated and control plots in **(A)** spring 2014 and **(B)** autumn 2015**. Bars from the same plot marked with the same letter are not significantly different (*P* > 0.05).

**FIGURE 7:**
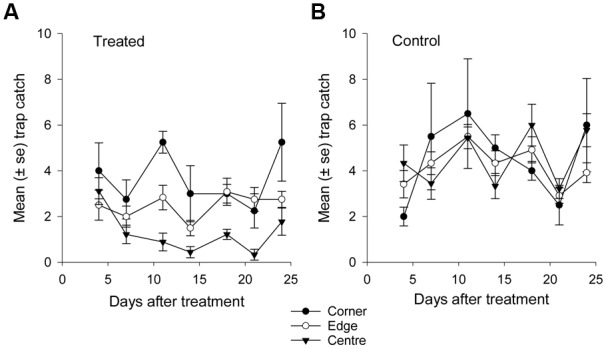
**Mean catch of African black beetles per pitfall trap in spring 2014 from corner to edge to center for 4–24 days after treatment in **(A)** treated and **(B)** control plots**.

More than 1100 beetles were caught in spring and the sex ratio was about 2:1 male:female (*P* = 0.01, **Table 2**). More beetles were caught in the control than the treated plots (*P* < 0.0001) and this treatment effect was consistent at all sample dates except the first (day 4 after treatment, *P* = 0.76; all other days, *P* ≤ 0.003; **Figure 5A**). There were strong spatial effects on trap catches but these effects differed between treated and control plots (significant treatment^∗^trap location interaction, *P* < 0.0001). The numbers of beetles caught in the treated plot declined from corner to edge to center but the control plot showed no significant spatial effects (**Figure 6A**). Corner trap catches were similar in treated and control plots (*P* = 0.28) but edge and center trap catches were lower in the treated plot than the control (*P* < 0.0001 for both comparisons). These spatial trends became apparent in the treated plot from the second sample date onward (day 7 after treatment, significant trap location^∗^day interaction, *P* < 0.0001, **Figure 7**).

**Table 2:** Total number of male and female beetles caught in the control and treated plots in spring 2014 and autumn 2015.

	Spring 2014			Autumn 2015		
Plot type	Male	Female	Total	Male	Female	Total
Treated	256	136	392	104	88	192
Control	470	292	762	166	136	302
Total	726	428	1154	270	224	494

#### Autumn 2015

**FIGURE 8:**
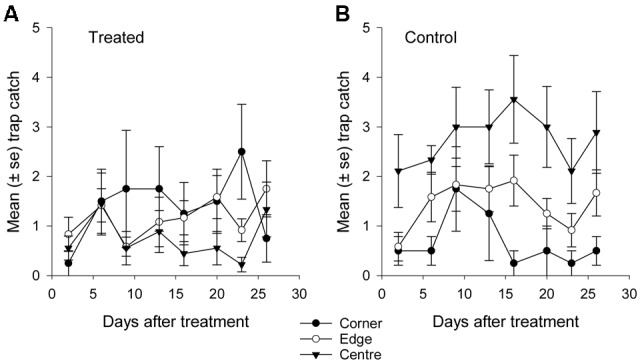
**Mean catch of African black beetles per pitfall trap in autumn 2015 from corner to edge to center for 2–26 days after treatment in **(A)** treated and **(B)** control plots**.

Fewer than 500 beetles were caught in autumn and the sex ratio was about 1:1 male:female (**Table 2**). More beetles were caught in the control than the treated plots (*P* < 0.001) but the treatment effect was less consistent across sample dates (days 9 and 16 after treatment, *P* ≤ 0.01; days 2 and 13 after treatment, *P* ≤ 0.09; all other days *P* ≥ 0.1, **Figure 5B**). Again, there were strong spatial effects on trap catches that differed between treated and control plots (significant treatment^∗^trap location interaction, *P* = 0.0006). The numbers of beetles caught in the treated plot tended to decline from corner to edge to center although only the corner traps captured significantly more beetles than the center (**Figure 6B**). There was an unexpectedly strong spatial effect in the control plot that was opposite to the pattern of the treated plot, i.e., trap catch increased from the corner to edge to center (**Figure 6B**). To compare spatial patterns between the two plots: more beetles were caught in the center of the control plot than the treated plot (*P* < 0.0001), similar numbers were caught in the edge traps of both plots (*P* = 0.35) and fewer beetles were caught in the corner traps of the control plot than the treated plot (*P* = 0.015). Spatial trends became apparent in the treated plot from the third sample date onward (day 9 after treatment, significant trap location^∗^day interaction, *P* < 0.0001, **Figure 8A**) but were seen in the control plot throughout the sampling period (**Figure 8B**).

### Mark–Release–Recapture (Spring 2014 and Autumn 2015)

Only marked males were recaptured in both spring and autumn (**Table 3**). In spring 11 marked beetles were recaptured (1.4% of marked beetles), including 1–2 individuals from each color group except silver, so recaptures were evenly spread across the two plots. The first marked beetle was collected 11 days after treatment and six marked beetles were collected on the last sample date, 24 days after treatment. The males were moving 0.45 ± 0.05 m/day (mean ± SE) in spring when the time elapsed between the release date and recapture dates is taken into account. Just one beetle with a blue marker was recaptured in autumn 23 days after treatment and approximately 12.8 m from its release point (= 0.55 m/day). No further analysis was attempted due to the low recapture rate in both seasons.

**Table 3:** Marked beetles recaptured in spring 2014.

Plot type	Color mark	Days until recapture	Minimum distance traveled (m)	Daily distance (m)
Treated	Dark green	14	7.07	0.51
	Purple	18	7.07	0.39
	Purple	21	15.81	0.75
	Orange	24	7.07	0.29
	Dark green	24	7.07	0.29
	Red	24	15.81	0.66
Control	Pink	11	7.07	0.64
	Yellow	14	7.07	0.51
	Yellow	24	7.07	0.29
	Light green	24	7.07	0.29
	Light green	24	7.07	0.29

*The minimum distance traveled was calculated as the shortest route from release point to recapture point for each color mark.*

## Discussion

Warmer temperatures increased walking by adult *H. arator* in Waikato and led to higher capture rates in pitfall traps, up to a maximum that presumably reflected the background population density and the catchment area of individual traps. Adult activity was also positively correlated with temperature in Tanzania (Abdallah et al., 2016). Higher surface activity relative to flight activity occurred in spring in Western Australia, but the reverse occurred in autumn (Matthiessen and Learmonth, 1998). Temperature variability will affect capture rates when monitoring field plot trials and probably accounts for some of the variation between sample dates seen here. While such variation is unlikely to change overall treatment effects, awareness of the prevailing weather conditions will assist interpretation of pitfall trap data for this pest.

Males were more active in spring, presumably while searching for mates, shifting to an approximately equal sex ratio in autumn when captured beetles are reproductively immature. Greater male activity was also seen in Australia during spring, but autumn catches were female-biased (Matthiessen and Learmonth, 1998). The reason for this difference in autumn captures is unclear. One possibility is the timing of the respective studies relative to the annual life cycle of *H. arator*. The Australian study continued trapping throughout most of the year. Perhaps trap catches in New Zealand shift to female dominated in late autumn, after this experiment ended.

The recapture rate for marked beetles was very low but did support the finding that males are more active than females in spring. A recent study used MRR on adult *H. arator* in Tanzanian maize crops with higher release numbers and achieved higher recapture rates, although recapture declined with increasing distance from the release point (Abdallah et al., 2016). The number of pitfall traps used was not reported, however, and the trap spacing did not follow a grid pattern so direct comparisons cannot be made easily between the two experiments. A longer trapping period after release may have increased recapture rates in this study, but the paddock was needed for grazing. A shorter distance between traps is also likely to increase recapture rates if MRR is used with *H. arator* again in New Zealand pastures.

The spatial analysis of trap catches suggested that adult *H. arator* were moving into the treated plot from the surrounding untreated area in both spring and autumn. Matthiessen (1999) inferred that adult beetles disperse in autumn from areas of high to low population density, leading to uniform spatial distributions for overwintering populations. Adult dispersal from untreated areas into treated field plots is a significant factor contributing to the failure of ‘pulse’ effect controls, e.g., insecticides (Eden et al., 2011). In contrast, open field plot experiments using endophyte varieties and similar plot sizes do report significant ongoing effects on *H. arator* populations (Thom et al., 2014; Barker et al., 2015). Endophytes provide (near) continuous expression of deterrent compounds, discouraging dispersal from untreated areas.

The most likely cause of spatial effects seen in the control plot in autumn was the placement of plots close to the paddock boundaries [as described in African Black Beetle Dispersal into Areas of Low Density (Spring 2014 and Autumn 2015)]. The control plot had the smallest area of surrounding paddock to act as a source of beetles compared to the treated plot in autumn and to both plots in spring. This demonstrates that placement of field plots relative to paddock boundaries and other landscape features can affect beetle movement.

Pitfall traps provide a measure of adult *H. arator* activity and therefore should be a useful tool to ensure optimal timing and greater efficacy of ‘pulse’ effect treatments used to protect pastures or crops. To prevent pasture damage from larvae over summer, treatments need to target African black beetle adults in spring before substantial oviposition occurs. For example, our results indicate late October would have been the optimum time for treatments to protect vulnerable new pastures in the study district. Pitfall traps also demonstrate spatial effects in field plots, and are an effective method for studying ground dispersal in this species, similar to other ground dwelling beetles (Noronha and Cloutier, 1999; Elek et al., 2014). For future insecticide experiments with *H. arator* larger plot sizes, more frequent monitoring after treatment, and consideration of spatial effects in the sampling design are all recommended so that treatment effects can be distinguished effectively from spatial effects due to beetle dispersal.

## Author Contributions

SM and PG designed the experiments; MH led the biopesticide development; SM, PG, RT, and DW set up the field experiments with DW and RT responsible for ongoing monitoring and data collection. CK analyzed the data and all authors contributed to data interpretation and writing.

## Conflict of Interest Statement

The authors declare that the research was conducted in the absence of any commercial or financial relationships that could be construed as a potential conflict of interest.

The reviewer AF and handling Editor declared their shared affiliation, and the handling Editor states that the process nevertheless met the standards of a fair and objective review.

## References

[B1] Abdallah M. Mwatawala M. W. Kudra A. B. (2016). Abundance and dispersal of *Heteronychus arator* (Coleoptera: Scarabaeidae) in maize fields under different fertilizer treatments. *SpringerPlus* 5:179 10.1186/s40064-016-1847-8 PMC4766157 27026875

[B2] Ball O. J. P. Miles C. O. Prestidge R. A. (1997). Ergopeptine alkaloids and *Neotyphodium* lolii-mediated resistance in perennial ryegrass against adult *Heteronychus arator* (Coleoptera: Scarabaeidae). *J. Econ. Entomol.* 90 1382–1391. 10.1093/jee/90.5.1382

[B3] Barker G. M. Patchett B. J. Cameron N. E. (2015). *Epichloë uncinata* infection and loline content afford *Festulolium* grasses protection from black beetle (*Heteronychus arator*). *N. Z. J. Agric. Res.* 58 35–56. 10.1080/00288233.2014.978480

[B4] Bell N. L. Townsend R. J. Popay A. J. Mercer C. F. Jackson T. A. (2011). “Black beetle: lessons from the past and options for the future,” in *Grassland Research and Practice Series No. 15* ed. Mercer C. F. (Dunedin: New Zealand Grassland Association) 119–124.

[B5] Bulinski J. Matthiessen J. N. (2002). Poor efficacy of the insecticide chlorpyrifos for the control of African black beetle (*Heteronychus arator*) in eucalypt plantations. *Crop Prot.* 21 621–627. 10.1016/S0261-2194(02)00012-1

[B6] Bulinski J. Matthiessen J. N. Alexander R. (2006). Development of a cost-effective, pesticide-free approach to managing African black beetle (*Heteronychus arator*) in Australian eucalyptus plantations. *Crop Prot.* 25 1161–1166. 10.1016/j.cropro.2005.12.006

[B7] Chapman R. B. (1984). “Pasture pests,” in *New Zealand Pest and Beneficial Insects* ed. Scott R. R. (Christchurch: Lincoln University College of Agriculture) 119–142.

[B8] Eden T. M. Gerard P. J. Wilson D. J. Addison P. J. (2011). Evaluation of spring and autumn applied insecticides for the control of black beetle. *N. Z. Plant Prot.* 64 63–67.

[B9] Elek Z. Drag L. Pokluda P. Cizek L. Berces S. (2014). Dispersal of individuals of the flightless grassland ground beetle, *Carabus hungaricus* (Coleoptera: Carabidae), in three populations and what they tell us about mobility estimates based on mark-recapture. *Eur. J. Entomol.* 111 663–668. 10.14411/eje.2014.080

[B10] Gerard P. J. Bell N. L. Eden T. M. King W. M. Mapp N. R. Pirie M. R. (2013). Influence of pasture renewal, soil factors and climate on black beetle abundance in Waikato and Bay of Plenty. *Proc. N. Z. Grass. Assoc.* 75 235–240.

[B11] Hardwick S. (2004). Colonisation of renovated pastures in Waikato by four coleopteran species. *N. Z. Plant Prot.* 57 304–309.

[B12] Hurst M. R. H. Becher S. A. Young S. D. Nelson T. L. Glare T. R. (2011a). *Yersinia entomophaga* sp. nov., isolated from the New Zealand grass grub *Costelytra zealandica*. *Int. J. Syst. Evol. Microbiol.* 61 844–849. 10.1099/ijs.0.024406-0 20495033

[B13] Hurst M. R. H. Rogers D. J. Wright D. A. Townsend R. J. Bruening R. Cole L. M. (2011b). Effect of the bacterium *Yersinia entomophaga* on adult bronze beetle. *N. Z. Plant Prot.* 64 209–214.

[B14] Hurst M. R. H. Swaminathan J. (2016). *Agricultural Composition. New Zealand Patent No. 716740.* Wellington: Intellectual Property Office.

[B15] King P. D. Mercer C. F. Meekings J. S. (1981). Ecology of black beetle, *Heteronychus arator* (Coleoptera: Scarabaeidae) — influence of pasture species on oviposition site preference. *N. Z. J. Zool.* 8 119–122.

[B16] Klingenberg M. D. Björklund N. Aukema B. H. (2010). Seeing the forest through the trees: differential dispersal of *Hylobius warreni* within modified forest habitats. *Environ. Entomol.* 39 898–906. 10.1603/EN08269 20550804

[B17] Matthiessen J. N. (1999). Late immature mortality is the major influence on reproductive success of African black beetle, *Heteronychus arator* (Fabricius) (Coleoptera: Scarabaeidae), in a Mediterranean-climate region of Australia. *Aust. J. Entomol.* 38 348–353. 10.1046/j.1440-6055.1999.00123.x

[B18] Matthiessen J. N. Learmonth S. E. (1995). Impact of the soil insects African black beetle, *Heteronychus arator* (Coleoptera: Scarabaeidae) and whitefringed weevil, *Graphognathus leucoloma* (Coleoptera: Curculionidae), on potatoes and effects of soil insecticide treatments in south-western Australia. *Bull. Entomol. Res.* 85 101–111. 10.1017/S0007485300052068

[B19] Matthiessen J. N. Learmonth S. E. (1998). Seasonally contrasting activity of African black beetle, *Heteronychus arator* (Coleoptera: Scarabaeidae): implications for populations, pest status and management. *Bull. Entomol. Res.* 88 443–450. 10.1017/S0007485300042188

[B20] Matthiessen J. N. Ridsdill-Smith T. J. (1991). Populations of African black beetle, *Heteronychus arator* (Coleoptera: Scarabaeidae) in a Mediterranean climate region of Australia. *Bull. Entomol. Res.* 81 85–91. 10.1017/S000748530005327X

[B21] Negro M. Casale A. Migliore L. Palestrini C. Rolando A. (2008). Habitat use and movement patterns in the endangered ground beetle species, *Carabus olympiae* (Coleoptera: Carabidae). *Eur. J. Entomol.* 105 105–112. 10.14411/eje.2008.015

[B22] NIWA (2015). *CliFlo: NIWA’s National Climate Database*. Available at: http://cliflo.niwa.co.nz/ [accessed March 11 2015]

[B23] Noronha C. Cloutier C. (1999). Ground and aerial movement of adult Colorado potato beetle (Coleoptera: Chrysomelidae) in a univoltine population. *Can. Entomol.* 131 521–538. 10.4039/Ent131521-4

[B24] Thom E. R. Popay A. J. Waugh C. D. Minneé E. M. K. (2014). Impact of novel endophytes in perennial ryegrass on herbage production and insect pests from pastures under dairy cow grazing in northern New Zealand. *Grass Forage Sci.* 69 191–204. 10.1080/00480169.2012.715379

[B25] Watson R. N. (1979). “Dispersal and distribution of *Heteronychus arator* in New Zealand (Coleoptera: Scarabaeidae),” in *Proceedings of the 2nd Australasian Conference on Grassland Invertebrate Ecology* Palmerston North 149–152.

[B26] Watson R. N. Marsden R. S. Townsend R. J. (1980). “Farm surveying of black beetle populations in spring as an indicator of larval populations in summer,” in *Proceedings of the 33rd New Zealand Weed and Pest Control Conference* Tauranga 144–147.

[B27] Winder L. (2004). Marking by abrasion or branding and recapturing carabid beetles in studies of their movement. *Int. J. Pest Manag.* 50 161–164. 10.1080/09670870410001731871

